# Informal Digital Information Use in Hospital Mass Casualty Incident Management During Wartime: Multicenter Cross-Sectional Questionnaire Study

**DOI:** 10.2196/109368

**Published:** 2026-11-13

**Authors:** Maya Benudiz, Noam Sabag, Aya Cohen, Nikol Zorkin, Michael Markovits, Eyal Hashavia, Michal Koren, Or Peretz, Shachar Shapira, Daniel Trotzky

**Affiliations:** 1 Gray Faculty of Medicine and Health Sciences, Tel Aviv University, Klachkin St, Tel Aviv, 6997801, Israel, +972 3-6409222; 2 Department of Social Work and Department of Psychology, The Hebrew University of Jerusalem, Jerusalem, Israel; 3 Division of Emergency Medicine and Emergency Preparedness, Tel Aviv Sourasky University Medical Center, affiliated to the Gray Faculty of Medical and Health Sciences, Tel Aviv University, Tel Aviv, Israel; 4 Department of Emergency Medicine, Tel Aviv Sourasky University Medical Center, affiliated to the Gray Faculty of Medical and Health Sciences, Tel Aviv University, Tel Aviv, Israel; 5 Division of Trauma, Department of Surgery, Tel Aviv Sourasky University Medical Center, affiliated to the Gray Faculty of Medical and Health Sciences, Tel Aviv University, Tel Aviv, Israel; 6 School of Industrial Engineering and Management, Shenkar - Engineering. Design. Art., Ramat Gan, Israel; 7 Management Wing, Sheba Medical Center, Ramat Gan, Israel; 8 Department of Emergency and Disaster Management, School of Public Health, Gray Faculty of Medical & Health Sciences, Tel Aviv University, Tel Aviv, Israel; 9 Medical Management, Tel Aviv Sourasky University Medical Center, affiliated to the Gray Faculty of Medical and Health Sciences, Tel Aviv University, Tel Aviv, Israel

**Keywords:** mass casualty incidents, social media, WhatsApp, Telegram, emergency department, trauma management, wartime, decision-making, digital communication

## Abstract

**Background:**

Managing mass casualty incidents (MCIs) during an active warfare environment presents unique challenges, including operating under extreme uncertainty. Traditionally, emergency communications rely on strict, hierarchical channels from prehospital services to receiving hospitals. However, informal digital communication streams, particularly instant messaging apps such as WhatsApp and Telegram, are increasingly used within medical workflows. Despite international interest in public social media during disasters, empirical research regarding how hospital leadership echelons use informal messaging tools for real-time operational and clinical decision-making during active warfare remains sparse.

**Objective:**

This study aimed to characterize the adoption of social media and instant messaging platforms as early information sources among hospital-based MCI managers during wartime and to evaluate their specific impact on real-time operational and clinical decision-making.

**Methods:**

A multicenter, cross-sectional study was conducted using a structured questionnaire distributed across all 24 national medical centers authorized to receive MCIs during wartime in Israel. Target respondents encompassed diverse leadership echelons, including emergency department directors, trauma service directors, trauma coordinators, charge nurses, emergency team members, and hospital executive leadership.

**Results:**

Overall, 76 medical professionals representing key decision-makers across national receiving centers completed the survey. WhatsApp emerged as the most frequently used platform (median 3.0, IQR 2.00‐4.00). Although these informal channels provided rapid initial intelligence, 65.3% (49/75) of respondents encountered false information during emergency events. Consequently, 81.3% (n=61) reported routinely verifying social media data against official channels before executing major actions. Multivariate regression analysis revealed distinct pathways of influence: higher platform use intensity was strongly and independently associated with enhanced situational awareness (*P*=.002) and greater operational decision-making influence (*P*=.02). In addition, participants’ level of trust and reliance was associated with clinical decisions (*P*=.01) and real-time team management (*P*=.03). Furthermore, active platform engagement during live threat conditions was positively correlated with heightened situational awareness during missile launch alerts (*P*<.001) and missile fall alerts (*P*<.001). Platform use and trust behaviors remained consistent across years of professional experience, qualifications, and executive roles.

**Conclusions:**

Informal digital platforms, dominated by WhatsApp, serve as vital, rapid early information channels for hospital MCI leadership during wartime emergencies. While use intensity directly enhances situational awareness and operational control during active missile threats, clinical and team management actions remain strictly contingent on trust. Given the high exposure to unverified data, formalizing structured verification protocols and incorporating informal digital communication strategies into hospital disaster preparedness frameworks are essential to optimize real-time decision-making in future large-scale health emergencies.

## Introduction

Management of mass casualty incidents (MCIs) in civilian settings, particularly during wartime, requires rapid decision-making under conditions of extreme uncertainty [1]. Traditionally, hospitals have relied on formal communication channels, primarily reports from prehospital emergency medical services, to guide preparedness and response [2]. However, over the past decade, the rapid expansion of digital communication has introduced social media as an additional tool for constructing an early operational picture [3]. The concept of free open access medical education has demonstrated the value of open digital platforms for knowledge sharing within the emergency medicine community, yet their real-time operational use during active events remains insufficiently characterized [3].

Emerging evidence suggests that social media platforms may function as “environmental sensors,” capable of detecting abnormal events prior to the arrival of official reports [2]. Analysis of user-generated content has been shown to provide early signals that may assist hospitals in filtering relevant information from background noise [2]. Furthermore, geospatial analysis of social media activity has enabled accurate localization of natural disasters and security-related incidents, highlighting their potential utility in early situational assessment [4]. This real-time engagement is particularly critical during active threats. The transition from “passive monitoring” to “active search” on social media during alerts has been identified as a key mechanism for maintaining operational continuity and situational awareness in high-stakes environments [5]. Currently, patterns of information dissemination during crises increasingly reflect a growing reliance on private and semiclosed messaging apps for real-time communication among both civilians and professional responders [6]. These platforms are often perceived as more immediate and accessible channels for situational updates, facilitating rapid information exchange during evolving incidents [6]. Beyond general situational awareness, studies further indicate that health care professionals are increasingly incorporating instant messaging services like WhatsApp into their clinical routines; these tools are frequently used for professional consultations and the swift transmission of patient-specific data, signifying a deep integration into modern medical workflows [7]. However, this integration is not uniform across the health care hierarchy; professional seniority and age serve as significant predictors of digital behavior. Younger clinicians and medical residents, often characterized by higher levels of digital literacy, tend to exhibit a greater frequency of social media and instant messaging use for professional purposes compared with their more senior counterparts [8]. Furthermore, the level of adoption and trust in specific digital channels is frequently role-dependent, influenced by the unique informational needs and perceived professional benefits inherent to different organizational echelons [8]. In addition to that, despite their perceived advantages in speed and accessibility, such channels are also associated with an elevated risk of misinformation and rumor propagation, reflecting broader challenges identified in the use of social media during crises [3,6].

The integration of social media–derived information is not merely observational but may have direct operational implications for hospital management [9,10]. Recent technological advances have enabled forms of “digital triage,” using images and videos from the scene to support early clinical assessment and resource allocation [9,10]. At the same time, a major challenge remains the validation of such information against official reports from emergency services to avoid both overpreparation and underresponse [2].

Despite the growing body of literature on social media in disaster response [3], a significant gap remains in understanding the specific tools and platforms used by senior emergency and trauma personnel during real-world wartime MCIs and how their use influences decision-making and operational performance. Israel represents a unique “natural laboratory” due to its extensive experience with civilian MCIs under conditions of ongoing wars. This study aims to address this gap by systematically characterizing which digital platforms—particularly Telegram and dedicated messaging groups—are used in practice, and how they influence decision-making processes within emergency departments during wartime.

## Methods

### Study Design

This study was a multicenter, cross-sectional study, integrating a quantitative survey (5-point Likert scale) with a qualitative analysis of open-ended responses to capture the complexities of real-time information processing.

Critically, the entirety of data collection was conducted contemporaneously in March 2026, strictly during the active, live phase of the escalating armed conflict involving the United States, Israel, and Iran, frequently referred to in US media as the “US-Israel-Iran War,” which featured an intensive missile offensive that commenced on February 28 and continued until a ceasefire was established on April 7, 2026. Capturing responses entirely within this operational window ensured that participant evaluations reflected authentic, real-time decision-making dynamics under immediate threat rather than post hoc or retrospective recall. Throughout this period, Israel was under a sustained missile offensive, resulting in numerous real-time MCIs that required immediate and complex operational responses from health care leadership. To target the specific MCI leadership echelons required for this study, the questionnaire was disseminated electronically through formal, national professional WhatsApp communities and established leadership forums, from 24 medical centers designated to receive patients during MCIs at the national level, including the formal networks of the Israeli Association for Emergency Medicine for emergency department directors and head nurses, the National Trauma Coordinators Network, the national emergency preparedness directors forum, the Israeli hospital executive leadership community, and the trauma unit directors network of the Israeli Association for Trauma and Emergency Surgery. These included specialized cohorts such as the “Forum of Acting Emergency Department Directors” and the “National Emergency Preparedness Officers Forum.” To ensure optimal participant engagement while minimizing digital survey fatigue, the questionnaire link was distributed a total of 2 times. The survey used a 5-point Likert scale (1=not at all, 5=to a great extent) to measure the frequency of social media use, levels of trust, and the resultant operational impact on leadership decision-making.

This study was conducted and reported in accordance with the STROBE (Strengthening the Reporting of Observational Studies in Epidemiology) reporting guideline for cross-sectional studies.

### Recruitment and Participants

#### Recruitment Setting and Target Population

The study targeted senior health care professionals and frontline MCI leadership echelons across all 24 designated nationwide acute-care receiving medical centers in Israel. Eligible participants comprised key operational and clinical decision-makers involved in real-time MCI management during active wartime conditions, including hospital executive directors, emergency department (ED) directors, ED head nurses, trauma unit directors, trauma coordinators, institutional emergency preparedness officers, and Ministry of Health emergency management representatives.

#### Systematic Recruitment Procedures

Participant recruitment was conducted through a systematic, multichannel electronic dissemination strategy using established, closed national professional networks and formal leadership forums. Specifically, the survey link was distributed through the Israeli Association for Emergency Medicine leadership networks (comprising the National Forum of Emergency Department Directors and Head Nurses), the National Trauma Coordinators Network, the National Emergency Preparedness Officers Forum, the Israeli Association for Trauma and Emergency Surgery, and the Israeli Hospital Executive Leadership Forum.

Dissemination was executed electronically via authenticated professional mobile networks (closed, verified WhatsApp communities representing these official leadership bodies). To maximize participation across all receiving trauma centers while preventing digital survey fatigue during ongoing operational strains, a 2-wave distribution protocol was implemented (an initial dissemination wave followed by a follow-up reminder). Participation was completely voluntary, anonymous, and uncompensated.

### Statistical Analysis

Statistical analyses were conducted on complete available cases. Ordinal Likert-type items were summarized using medians and IQRs; continuous or derived scores were summarized using means and SDs where appropriate. Within-person platform-use comparisons were evaluated using paired nonparametric tests with Holm correction, and bivariate associations between composite predictors and outcomes were screened using Spearman rank correlations with Benjamini-Hochberg false discovery rate (FDR) correction.

For the additional analyses requested for the current revision, years of experience were examined against platform-use and trust items using Spearman rank correlations. Differences by professional qualification and current role were examined using Kruskal-Wallis tests; because several qualification categories were sparse, qualification categories were collapsed into registered nurse, physician, and other for this secondary analysis. The association between the availability of sufficient verification tools and exposure to false information was evaluated using the Fisher exact test, and associations between verification tools and ordinal decision influence items were evaluated using Mann-Whitney *U* tests. Associations between active search during missile launch or missile fall alerts and situational awareness were evaluated using Spearman rank correlations. These secondary analyses were considered exploratory, 2-sided, and interpreted with FDR-adjusted *P* values within each analysis family.

Adjusted cumulative logit models were fitted for manuscript-reportable ordinal outcomes using use intensity and trust and reliance as primary predictors, with experience and professional background variables included where estimable. Open-ended responses were summarized descriptively and used only as illustrative context rather than as a standalone qualitative strand.

### Ethical Considerations

The study was approved by the Tel Aviv Sourasky University Medical Center Institutional Review Board (approval ASAF-0172‐26) and conducted in accordance with the ethical principles of the Declaration of Helsinki and its later amendments. The requirement for written informed consent was waived by the institutional review board, and completion of the questionnaire was considered evidence of informed consent. The research instrument was developed and managed using the REDCap platform, a secure, web-based software application designed to support data capture and metadata-driven workflows for clinical research. All data were collected and stored anonymously to ensure participant privacy and confidentiality. No financial or other compensation was provided to participants.

## Results

### Sample Characteristics

The analytic sample comprised 76 respondents (Table 1). The largest professional qualification groups were physician (41/76, 53.9%) and registered nurse (25/76, 32.9%). The largest current role groups were hospital executive leadership (19/76, 25.0%) and ED director (12/76, 15.8%). The median experience in the current role was 7.0 (IQR 3.0‐13.0) years. Overall, 81.3% (61/75) reported confirming social media information against official sources before taking major action, and 65.3% (49/75) reported encountering false information during emergencies.

**Table 1.** Demographic, professional, and operational characteristics of hospital leadership respondents across 24 national mass casualty incident–receiving medical centers in Israel during active wartime (March 2026; N=76).

Characteristic	Participants, n (%)
Analytic sample	
Respondents	76 (100)
Professional qualification	
Registered nurse	25 (32.9)
Physician	41 (53.9)
Clinical specialist	5 (6.6)
Physician fellow	1 (1.3)
Paramedic	1 (1.3)
Other	3 (3.9)
Current role	
Emergency department director	12 (15.8)
Trauma service director	7 (9.2)
Trauma coordinator	3 (3.9)
Charge nurse	10 (13.2)
Emergency team member	12 (15.8)
Hospital executive leadership	19 (25)
Other	13 (17.1)
Experience group (years)	
0‐5	29 (44.6)
6‐10	14 (21.5)
11‐20	14 (21.5)
≥21	8 (12.3)
Has sufficient tools to confirm information	
Yes	24 (31.6)
No	52 (68.4)
Confirms information against official sources before action	
Yes	61 (81.3)
No	14 (18.7)
Encountered false information	
Yes	49 (65.3)
No	26 (34.7)

### Platform Use, Trust, and Verification Behavior

WhatsApp was the most frequently used platform (median 3.0, IQR 2.00‐4.00), followed by news channels (median 2.0, IQR 1.00‐3.00), whereas Telegram use was lower (median 1.0, IQR 1.00‐3.00; Table 2). All pairwise platform comparisons were statistically significant after correction except Telegram versus news channels. Among Telegram users, news channels and real-time field security channels were the most endorsed channel types.

**Table 2.** Descriptive metrics of digital platform use, behavioral trust, and perceived influence on operational and clinical decision-making among hospital mass casualty incident (MCI) commanders during wartime in Israel (March 2026; N=76).

Measure	Median (IQR)	Mean (SD)
Behavior and exposure (n=76)		
Social media as a useful tool during emergencies	3.0 (2.00‐4.00)	2.72 (1.26)
Exposure to social media information during MCI management	2.0 (1.00‐3.25)	2.43 (1.31)
Active search during missile launch alerts	3.0 (2.00‐4.00)	2.82 (1.23)
Active search during missile fall alerts	3.0 (2.00‐4.00)	3.13 (1.23)
Social media precedes official reporting	2.0 (1.00‐3.00)	2.39 (1.14)
Platform use frequency		
Telegram (n=76)	1.0 (1.00‐3.00)	1.95 (1.23)
WhatsApp (n=75)	3.0 (2.00‐4.00)	3.15 (1.36)
Facebook and X (n=76)	1.0 (1.00‐2.00)	1.53 (0.93)
News channels (n=75)	2.0 (1.00‐3.00)	2.32 (1.22)
Trust and reliance		
Trust in Telegram (n=75)	3.0 (1.50‐3.00)	2.52 (1.16)
Trust in WhatsApp (n=76)	3.0 (2.00‐3.00)	2.83 (0.82)
Trust in social media videos (n=74)	3.0 (2.00‐3.00)	2.53 (0.92)
Trust in news channels (n=76)	3.5 (3.00‐4.00)	3.41 (0.70)
Reliance on EMS^a^ groups (n=75)	4.0 (2.00‐4.00)	3.31 (1.23)
Perceived influence		
Helps form situational awareness (n=75)	2.0 (2.00‐3.00)	2.37 (0.88)
Influence on clinical decisions (n=74)	2.0 (1.00‐2.00)	1.82 (0.83)
Influence on operational decisions (n=74)	2.0 (1.00‐3.00)	2.15 (0.93)
Influence on MCI announcement decisions (n=73)	2.0 (1.00‐2.00)	1.66 (0.71)
Influence on team management during MCI (n=72)	2.0 (1.00‐3.00)	2.12 (1.02)

a EMS: emergency medical services.

Respondents rated social media as moderately helpful for situational awareness (median 2.0, IQR 2.00‐3.00). Perceived influence was modest for clinical decisions (median 2.0, IQR 1.00‐2.00) and operational decisions (median 2.0, IQR 1.00‐3.00), lowest for MCI announcement decisions (median 2.0, IQR 1.00‐2.00), and somewhat higher for team management during an MCI (median 2.0, IQR 1.00‐3.00).

### Scale Performance and Bivariate Associations

The use intensity (α=0.741), trust and reliance (α=0.653), and decision influence (α=0.821) composites showed acceptable-to-good internal consistency. In FDR-adjusted screening, higher use intensity was associated with greater situational-awareness ratings (ρ=0.674; FDR *P*<.001) and greater operational-decision influence (ρ=0.672; FDR *P*<.001) (Figure 1).

**Figure 1.**
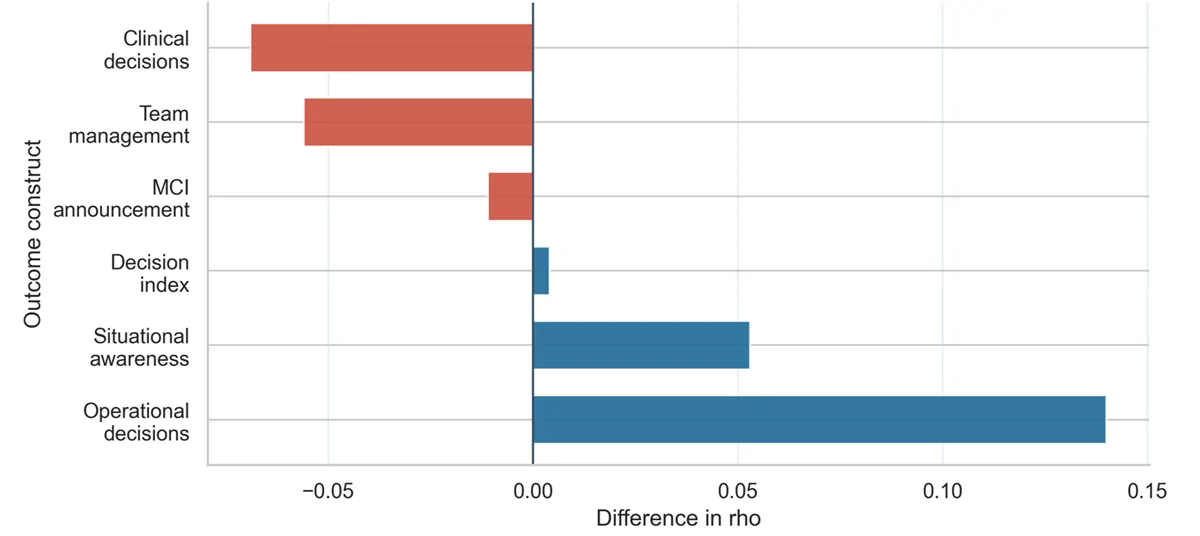
Differential bivariate correlation strengths (Δ Spearman ρ) of digital platform use intensity versus trust and reliance across situational awareness and emergency decision-making domains among Israeli hospital mass casualty incident (MCI) leadership during active wartime (March 2026; N=76; multicenter cross-sectional study). Bars extending to the right (blue) indicate outcomes more strongly correlated with platform use intensity, while bars extending to the left (red) indicate outcomes more strongly correlated with trust and reliance.

Higher levels of trust and reliance were associated with greater clinical decision influence (ρ=0.637, FDR *P*<.001) and with the composite decision influence index (ρ=0.621, FDR *P*<.001; Table 3). To summarize the relative strength of the 2 composites per outcome, we plotted the difference between Spearman correlations (use intensity index minus trust and reliance index). The overall visual pattern was consistent across outcomes: use intensity aligned most strongly with situational awareness and operational decisions, whereas trust and reliance aligned more strongly with clinical decisions and the overall decision influence composite.

**Table 3.** Bivariate Spearman rank correlations between composite digital predictors (use intensity and trust and reliance) and situational awareness and decision-making outcomes among hospital mass casualty incident (MCI) commanders in Israel (March 2026; N=76).

Outcome	Use intensity, ρ	FDR^a^-adjusted *P* value	Trust and reliance, ρ	Trust and reliance FDR-adjusted *P* value
Helps form situational awareness	0.674	<.001	0.621	<.001
Influence on clinical decisions	0.568	<.001	0.637	<.001
Influence on operational decisions	0.672	<.001	0.532	<.001
Influence on MCI announcement decisions	0.352	.007	0.363	.005
Influence on team management during MCI	0.377	.004	0.433	<.001
Decision influence index	0.625	<.001	0.621	<.001

a FDR: false discovery rate.

Additional secondary analyses addressed experience, professional role, verification capacity, and active searching (Table 1 and Multimedia Appendix 1). Years of experience were not significantly associated with individual platform use or trust items after FDR adjustment; the largest uncorrected signal was a weak inverse association with WhatsApp use frequency (ρ=-0.266; *P*=.03; FDR-adjusted *P*=.22). Professional qualification and current role also showed no significant differences across use and trust items after FDR adjustment, although current role differed in Telegram trust before multiplicity correction (Kruskal-Wallis H=14.350; *P*=.03; FDR-adjusted *P*=.21).

Reporting sufficient tools to confirm information was not associated with having encountered false information (Fisher exact test: odds ratio [OR]=1.091; *P>*.99), and it was not significantly associated with situational-awareness or decision influence items after FDR adjustment. In contrast, active search during alerts showed consistent positive associations with situational awareness, both during missile launch alerts (ρ=0.560; FDR-adjusted *P*<.001) and missile fall alerts (ρ=0.439; FDR-adjusted *P*<.001).

### Adjusted Ordinal Regression Models

Figure 2 shows, for each outcome, the adjusted OR and 95% CI for a 1-unit increase in the use intensity composite (left panel) and the trust and reliance composite (right panel), with estimates aligned on a logarithmic axis and a reference line at no effect (OR=1).

**Figure 2.**
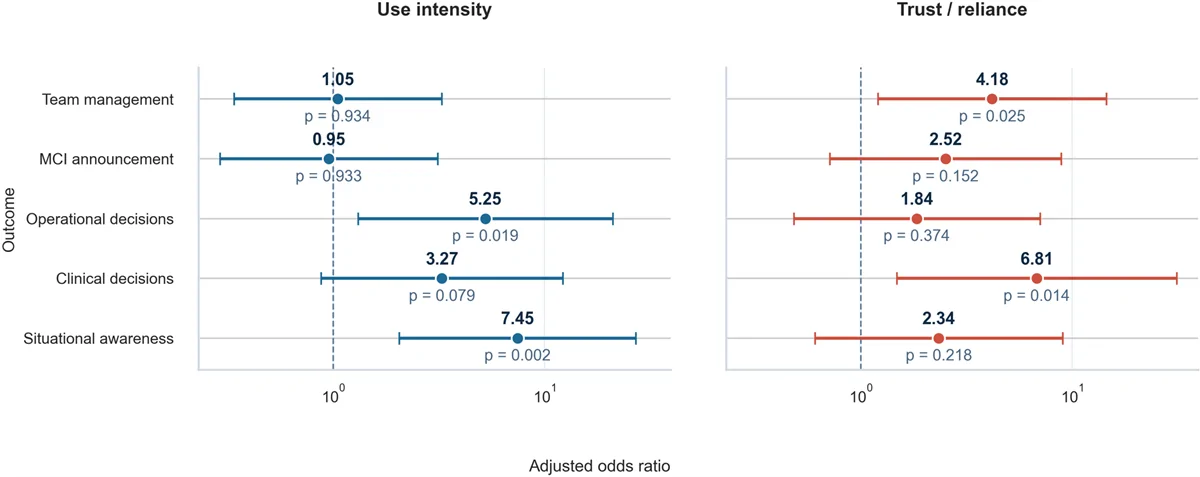
Adjusted odds ratios (aOR) and 95% CIs from multivariable ordinal logistic regression models predicting situational awareness, clinical, operational, and organizational outcomes from digital use intensity (blue) and trust and reliance (red) among hospital leadership during wartime in Israel (March 2026; N=76; national multicenter survey). Dashed vertical lines indicate the null effect (aOR=1.0). Estimates are adjusted for professional experience and leadership role. MCI: mass casualty incident.

The detailed numerical measures for ordinal models are summarized in Table 4. Use intensity remained the clearest independent correlate of situational awareness (OR 7.452, 95% CI 2.053‐27.056; *P*=.002), whereas trust and reliance did not add independent signal (OR 2.337, 95% CI 0.606‐9.017; *P*=.22). By contrast, trust and reliance was the dominant correlate of clinical decision influence (OR 6.811, 95% CI 1.481‐31.326; *P*=.01), while use intensity did not reach the conventional significance threshold (OR 3.267, 95% CI 0.873‐12.221; *P*=.08). Use intensity also showed the clearest adjusted association with operational-decision influence (OR 5.253, 95% CI 1.307‐21.109; *P*=.02), whereas trust and reliance was not independently associated (OR 1.840, 95% CI 0.48‐7.055; *P*=.37). Trust and reliance again provided the clearest adjusted signal for team management influence (OR 4.182, 95% CI 1.201‐14.563; *P*=.03), whereas use intensity was not independently associated (OR 1.049, 95% CI 0.338‐3.255; *P*=.93). For MCI announcement decisions, neither use intensity (OR 0.950, 95% CI 0.29‐3.116; *P*=.93) nor trust and reliance (OR 2.519, 95% CI 0.712‐8.905; *P*=.15) showed a clearly independent association.

**Table 4.** Multivariable cumulative logit ordinal regression models evaluating the independent effects of platform use intensity and trust and reliance on decision-making dimensions during wartime hospital mass casualty incident (MCI) management in Israel (March 2026; N=76).

Predictor	Odds ratio (95% CI)	*P* value
Helps form situational awareness		
Use intensity score	7.452 (2.053‐27.056)	.002
Trust and reliance index	2.337 (0.606‐9.017)	.22
Influence on clinical decisions		
Use intensity score	3.267 (0.873‐12.221)	.08
Trust and reliance index	6.811 (1.481‐31.326)	.01
Influence on operational decisions		
Use intensity score	5.253 (1.307‐21.109)	.02
Trust and reliance index	1.840 (0.48‐7.055)	.37
Influence on MCI announcement decisions		
Use intensity score	0.950 (0.29‐3.116)	.93
Trust and reliance index	2.519 (0.712‐8.905)	.15
Influence on team management during MCI		
Use intensity score	1.049 (0.338‐3.255)	.93
Trust and reliance index	4.182 (1.201‐14.563)	.03

A 2-profile exploratory clustering solution separated lower-engagement and lower-trust respondents from higher-engagement and higher-trust respondents; situational-awareness ratings differed across profiles (Kruskal-Wallis statistic 30.265; *P*<.001), and the decision influence composite differed across profiles (Kruskal-Wallis statistic 20.538; *P*<.001).

## Discussion

### Principal Results

This study evaluated how hospital MCI leaders used informal digital platforms such as WhatsApp during wartime to guide real-time operational and clinical decisions. Our analysis revealed that platform use intensity directly enhanced situational awareness and operational decision-making. Conversely, clinical decision-making and real-time team management were strictly contingent upon participants’ level of trust and reliance on the information source.

The preliminary findings highlight a phenomenon that can be termed “informal tactical triggering.” This concept describes a scenario wherein rapid, informal information, such as a Telegram post or a WhatsApp video, reaches managers or decision-makers and acts as an operational trigger that jumpstarts the system into action well before official reports are received. A critical insight from the data suggests that during wartime, the initial data received via these platforms serve as a vital sensor for the system. Another clear signal from our analysis was behavioral: respondents who actively searched for information during missile launch and missile fall alerts reported higher situational awareness ratings.

Moreover, our secondary analysis revealed that professional experience, background, and qualifications were not significantly associated with informal platform use or trust patterns, presenting a divergence from established literature. Historically, digital health research suggests a pronounced generational and seniority gap in health care settings. For instance, Farsi (2021) [8] documented that senior or more experienced clinicians consistently exhibit greater skepticism, lower adoption rates, and heightened risk aversion regarding social media integration compared with their junior counterparts. In contrast, our findings demonstrate a uniform reliance on informal communication streams across all professional echelons and age groups during wartime MCIs. This phenomenon may indicate that under conditions of acute operational stress and extreme uncertainty, such as the real-time threats of an active missile offensive, traditional digital divides dissolve. During emergencies, the critical need for rapid situational awareness and informal tactical triggering supersedes individual personal and professional habits, thereby transforming informal platforms from a generational preference into a universal and operational necessity.

Furthermore, our multivariable ordinal regression models delineate a fundamental operational dichotomy: platform use intensity independently governs situational awareness and macrolevel operational decisions, whereas clinical decisions and direct team management are strictly mediated by subjective trust. This dissociation reflects the practical mechanics of how emergency leaders operate under extreme uncertainty. Macrolevel logistics and system priming, such as clearing hallway bottlenecks, reserving operating suites, or holding off-duty personnel on standby, carry an asymmetric risk profile in which early preparation is cheap, but unpreparedness is catastrophic. Consequently, commanders leverage raw, low-trust visual streams (eg, unverified scene footage) as an early “sensor” to prime physical capacity. In stark contrast, microlevel clinical execution, such as deploying invasive trauma teams, entails immediate patient safety risks. Hospital commanders therefore apply a rigorous cognitive threshold: informal digital intelligence triggers operational posture, but definitive clinical intervention requires high-trust validation and institutional corroboration.

### Key Thematic Framework

#### The Digital Paradox

There is a notable contradiction between the reported low trust in social media content—frequently characterized by respondents as “mostly fake” or “sensationalized”—and its high operational utility. Despite the prevalence of misinformation, the visual nature of the data (videos and photos from the scene) allows for a rapid assessment of the event’s scale.

#### The Social Media Sensor

Rather than replacing formal reports, social media acts as a “system trigger.” Managers use informal reports to proactively initiate contact with Magen David Adom (MDA, Israel's national emergency medical service), effectively narrowing the information gap and allowing for an earlier declaration of an MCI.

#### Operational Readiness

Preliminary results suggest that visual information from the scene directly influences the decision to “prime” the system—preparing trauma bays and adjusting staffing levels—prior to the arrival of the first ambulance.

#### Verification Protocols

A recurring theme is the necessity of a “verification loop,” where informal digital alerts are immediately cross-referenced with formal emergency medical services (EMS) radio traffic to prevent overtriage or false alarms.

### Limitations

This study has several limitations that should be considered when interpreting the findings.

First, the overall sample size is relatively small (N=76), and several role categories contained few participants. This may limit the statistical power of certain subgroup analyses and requires that these subgroup findings remain exploratory, which may also reduce broader generalizability. Relatedly, because the survey was distributed electronically across established, closed national leadership forums and professional group channels with overlapping memberships, the exact aggregate denominator of unique individuals who viewed the electronic invitation could not be determined with complete precision, precluding the calculation of a conventional response rate. However, both methodological constraints must be contextualized within the unique structural scale of the Israeli health care system. Nationwide, there are only 24 general hospitals with EDs designated as receiving sites during MCIs. Because the national pool of specialized trauma directors, ED managers, emergency preparedness officers, and executive leaders is inherently constrained (estimated at approximately 120‐150 eligible frontline commanders nationwide), a sample of 76 respondents does not merely represent a minor cohort, but rather constitutes a substantial and highly representative proportion of the country’s frontline wartime medical leadership, achieving an estimated substantive national coverage of more than 50%. Furthermore, although the overall cohort (N=76) represents a substantial proportion of frontline MCI leadership across all 24 authorized receiving hospitals, some specialized subgroups were represented by very small numbers of participants, including physician fellows (n=1), paramedics (n=1), and trauma coordinators (n=3). Therefore, subgroup comparisons, including nonsignificant Kruskal-Wallis tests, should be interpreted with caution, as the small subgroup sizes limit statistical power and may increase the risk of type II error. These analyses should therefore be considered exploratory, and nonsignificant findings should not be interpreted as evidence that meaningful differences between professional roles do not exist.

Second, because the questionnaire was distributed primarily through professional WhatsApp networks, this recruitment method could introduce selection bias toward respondents who are inherently more active on WhatsApp or favorable toward digital instant messaging. However, this potential bias is mitigated by the fact that these WhatsApp networks serve as the official, nationwide operational channels for Israeli health care leadership during emergencies, making them the most direct and realistic medium to reach this specific target population.

Finally, our analysis showed that having personal verification tools did not protect respondents from being exposed to false information. While this might seem like a limitation in our findings, it actually reveals an important insight: verifying real-time digital information cannot rely solely on the skills or tools of an individual clinician. Instead, it demonstrates that data verification must be built directly into hospital operations as an official organizational protocol.

### Conclusions

The integration of informal digital intelligence into hospital MCI protocols represents an operational shift in emergency management. While formal prehospital channels remain the gold standard for verified data, informal digital streams, dominated by WhatsApp, have become indispensable environmental sensors for rapid situational awareness. Crucially, this study demonstrates that reliance on informal platforms is a universal operational reality that spans all age groups, professional roles, and seniority levels during acute wartime stress.

However, this reliance uncovers a critical vulnerability termed the verification paradox. While 81.3% (61/75) of decision-makers actively attempted to confirm digital intelligence before taking major actions, 68.4% (52/76) reported lacking adequate tools to verify information in real time. To bridge this systemic gap, health care systems should consider shifting from relying on individual ad hoc verification to establishing formal institutional protocols. Furthermore, disaster management frameworks could benefit from institutionalizing verification loops, which are structured operational protocols that rapidly cross-reference informal visual data from field scenes with incoming EMS radio traffic before committing clinical resources or over-triaging.

Finally, future disaster preparedness should address the inherent technical fragilities of relying on instant messaging apps. During kinetic warfare or large-scale mass casualty events, physical damage to cellular infrastructure, network congestion, or deliberate cyberattacks and communications blackouts pose risks to operational continuity. Emergency planners are therefore advised to ensure technical redundancy by integrating localized backup communication networks alongside digital intelligence protocols. A thorough understanding of informal digital channels, along with their strategic integration as operational triggers, can significantly enhance hospital readiness, provided it is supported by institutional verification frameworks and resilient communication infrastructure.

## Supplementary material

10.2196/109368 Multimedia Appendix 1 Supplementary tables of secondary subgroup analyses (experience, professional role, and verification capacity) and the complete study questionnaire.

## Abbreviations

ED: emergency department
EMS: emergency medical services
FDR: false discovery rate
MCI: mass casualty incident
MDA: Magen David Adom
STROBE: Strengthening the Reporting of Observational Studies in Epidemiology
