# Supplementary material for: Informal Digital Information Use in Hospital Mass Casualty Incident Management During Wartime: Multicenter Cross-Sectional Questionnaire Study
Source: J Med Internet Res. 2026 Nov 13;28:e109368. doi: 10.2196/109368 (PMC13761662; doi:10.2196/109368)
Supplement: Supplementary file 1 [file jmir-v28-e109368-s001.docx]

Table S1. Analysis of experience, professional qualification, and current role with platform-use and trust items.

| **Analysis** | **Item** | **n** | **Test** | **Effect** | **p** | **FDR q** |
| --- | --- | --- | --- | --- | --- | --- |
| Experience (years) | Telegram use frequency | 65 | Spearman rho | -0.218 | 0.081 | 0.227 |
| Experience (years) | WhatsApp use frequency | 64 | Spearman rho | -0.266 | 0.034 | 0.227 |
| Experience (years) | Facebook/X use frequency | 65 | Spearman rho | -0.191 | 0.128 | 0.227 |
| Experience (years) | News-channel use frequency | 65 | Spearman rho | -0.069 | 0.583 | 0.583 |
| Experience (years) | Trust in Telegram | 64 | Spearman rho | -0.203 | 0.109 | 0.227 |
| Experience (years) | Trust in WhatsApp | 65 | Spearman rho | -0.159 | 0.206 | 0.274 |
| Experience (years) | Trust in social-media videos | 63 | Spearman rho | -0.187 | 0.142 | 0.227 |
| Experience (years) | Trust in news channels | 65 | Spearman rho | 0.110 | 0.383 | 0.438 |
| Qualification (nurse/physician/other) | Telegram use frequency | 76 | Kruskal-Wallis H | 0.144 | 0.931 | 0.931 |
| Qualification (nurse/physician/other) | WhatsApp use frequency | 75 | Kruskal-Wallis H | 1.538 | 0.464 | 0.618 |
| Qualification (nurse/physician/other) | Facebook/X use frequency | 76 | Kruskal-Wallis H | 4.319 | 0.115 | 0.461 |
| Qualification (nurse/physician/other) | News-channel use frequency | 75 | Kruskal-Wallis H | 2.121 | 0.346 | 0.587 |
| Qualification (nurse/physician/other) | Trust in Telegram | 75 | Kruskal-Wallis H | 2.004 | 0.367 | 0.587 |
| Qualification (nurse/physician/other) | Trust in WhatsApp | 76 | Kruskal-Wallis H | 5.044 | 0.080 | 0.461 |
| Qualification (nurse/physician/other) | Trust in social-media videos | 74 | Kruskal-Wallis H | 2.661 | 0.264 | 0.587 |
| Qualification (nurse/physician/other) | Trust in news channels | 76 | Kruskal-Wallis H | 0.959 | 0.619 | 0.707 |
| Current role | Telegram use frequency | 76 | Kruskal-Wallis H | 9.532 | 0.146 | 0.389 |
| Current role | WhatsApp use frequency | 75 | Kruskal-Wallis H | 10.431 | 0.108 | 0.389 |
| Current role | Facebook/X use frequency | 76 | Kruskal-Wallis H | 3.874 | 0.694 | 0.793 |
| Current role | News-channel use frequency | 75 | Kruskal-Wallis H | 7.769 | 0.256 | 0.450 |
| Current role | Trust in Telegram | 75 | Kruskal-Wallis H | 14.350 | 0.026 | 0.208 |
| Current role | Trust in WhatsApp | 76 | Kruskal-Wallis H | 7.450 | 0.281 | 0.450 |
| Current role | Trust in social-media videos | 74 | Kruskal-Wallis H | 4.471 | 0.613 | 0.793 |
| Current role | Trust in news channels | 76 | Kruskal-Wallis H | 1.895 | 0.929 | 0.929 |

Table S2. Verification, misinformation, decision-influence, and active-search analysis.

| **Analysis** | **Item** | **n** | **Test** | **Effect** | **p** | **FDR q** |
| --- | --- | --- | --- | --- | --- | --- |
| Verification tools vs false information | Encountered false information | 75 | Fisher exact OR | 1.091 | 1.000 | 1.000 |
| Verification tools vs influence items | Situational awareness | 75 | Mann-Whitney U | 724.500 | 0.178 | 0.296 |
| Verification tools vs influence items | Clinical decisions | 74 | Mann-Whitney U | 721.000 | 0.094 | 0.235 |
| Verification tools vs influence items | Operational decisions | 74 | Mann-Whitney U | 754.000 | 0.063 | 0.235 |
| Verification tools vs influence items | MCI announcement decisions | 73 | Mann-Whitney U | 598.500 | 0.764 | 0.818 |
| Verification tools vs influence items | Team management during MCI | 72 | Mann-Whitney U | 568.500 | 0.818 | 0.818 |
| Active search vs situational awareness | Active search during missile-launch alert | 75 | Spearman rho | 0.560 | <0.001 | <0.001 |
| Active search vs situational awareness | Active search during missile-fall alert | 75 | Spearman rho | 0.439 | <0.001 | <0.001 |

## Study Questionnaire

**1. What is your professional qualification? ***

- Registered Nurse
- Physician
- Clinical Nurse Specialist / Nurse Practitioner
- Physician Assistant (PA)
- Paramedic
- EMT / Medic
- Other (please specify in the next section)

**2. What is your current organizational role? ***

- Emergency Department (ED) Director
- Trauma Center Director
- Trauma Coordinator
- Head Nurse / Charge Nurse
- Emergency Preparedness Committee Member
- Hospital Executive Leadership
- Other (please specify in the next section)

**3. How many years of experience do you have in your current role? ***

[Open-ended text/numerical response]

**4. To what extent do you view social media as a useful tool during emergencies?**

- Not at all
- To a small extent
- To a moderate extent
- To a great extent
- To a very great extent

**5. To what extent are you exposed to information obtained from social media while managing a Mass Casualty Incident (MCI)?**

- Not at all
- To a small extent
- To a moderate extent
- To a great extent
- To a very great extent

**6. To what extent do you actively search for information on social media when a missile launch alert is received?**

- Not at all
- To a small extent
- To a moderate extent
- To a great extent
- To a very great extent

**7. To what extent do you actively search for information on social media when a missile impact/fall alert is received?**

- Not at all
- To a small extent
- To a moderate extent
- To a great extent
- To a very great extent

**8. How frequently do you use Telegram to obtain initial information during MCI management?**

- Never
- Rarely
- Sometimes
- Often
- Always

**9. How frequently do you use WhatsApp to obtain initial information during MCI management?**

- Never
- Rarely
- Sometimes
- Often
- Always

**10. How frequently do you use public social media platforms (e.g., Facebook, X/Twitter) during MCI management?**

- Never
- Rarely
- Sometimes
- Often
- Always

**11. How frequently do you watch television news broadcasts or live streaming during your work throughout an event?**

- Never
- Rarely
- Sometimes
- Often
- Always

**12. To what extent do you rely on professional operational channels (e.g., Magen David Adom [MDA], United Hatzalah, etc.)?**

- Not at all
- To a small extent
- To a moderate extent
- To a great extent
- To a very great extent

**13. To what extent do you receive initial information regarding an MCI from social media before official reports are disseminated?**

- Not at all
- To a small extent
- To a moderate extent
- To a great extent
- To a very great extent

**14. Rate your level of trust in the information received from the Telegram channels you utilize.**

- No trust at all
- Low trust
- Moderate trust
- High trust
- Very high trust

**15. Among the following types of Telegram channels, which do you utilize? (Select all that apply):**

- Official security update channels
- General news channels
- Real-time field-level security update channels
- Video aggregate channels from conflict zones

**16. Among the following types of Telegram channels, which type do you consider to be the most reliable?**

- Official security update channels
- General news channels
- Real-time field-level security update channels
- Video aggregate channels from conflict zones

**17. Rate your level of trust in the information received from WhatsApp groups.**

- No trust at all
- Low trust
- Moderate trust
- High trust
- Very high trust

**18. Rate your level of trust in videos distributed across social media platforms.**

- No trust at all
- Low trust
- Moderate trust
- High trust
- Very high trust

**19. Rate your level of trust in information originating from broadcast media (radio and television news).**

- No trust at all
- Low trust
- Moderate trust
- High trust
- Very high trust

**20. Do you possess sufficient tools to identify misinformation or false information on social networks in real time?**

- Yes
- No

**21. Do you make sure to confirm all information obtained from social media with an official authority (e.g., EMS/Military) before taking major action during emergencies?**

- Yes
- No

**22. Have you ever encountered false information or fake news through social media platforms?**

- Yes
- No

**23. To what extent does information from social media assist you in formulating a situational awareness assessment during emergencies?**

- Not at all
- To a small extent
- To a moderate extent
- To a great extent
- To a very great extent

**24. To what extent are you influenced on a clinical level by information received from social media during emergencies?**

- Not at all
- To a small extent
- To a moderate extent
- To a great extent
- To a very great extent

**25. To what extent are you influenced on an operational level by information received from social media during emergencies?**

- Not at all
- To a small extent
- To a moderate extent
- To a great extent
- To a very great extent

**26. To what extent are you influenced by social media when deciding whether to declare an MCI status?**

- Not at all
- To a small extent
- To a moderate extent
- To a great extent
- To a very great extent

**27. To what extent are you influenced by social media when deciding whether to alert or prep/staff clinical teams during an MCI?**

- Not at all
- To a small extent
- To a moderate extent
- To a great extent
- To a very great extent

**28. What insights do you gain from information received during an MCI via social networks, and how do they impact your management and operational handling of the situation?**

[Open-ended text response]

**29. Do you have any additional insights regarding the impact of social media information during an MCI that was not addressed in this questionnaire?**

[Open-ended text response]

** Denotes a mandatory question (Must provide value).*
